# From Prostate to Bone: Key Players in Prostate Cancer Bone Metastasis

**DOI:** 10.3390/cancers3010478

**Published:** 2011-01-27

**Authors:** Megan N. Thobe, Robert J. Clark, Russell O. Bainer, Sandip M. Prasad, Carrie W. Rinker-Schaeffer

**Affiliations:** 1 Section of Urology, Department of Surgery, The University of Chicago, Chicago, IL 60637, USA; E-Mails: Thobemn@uchicago.edu (M.N.T.); sprasad1@surgery.bsd.uchicago.edu (S.M.P.); 2 Department of Molecular Pathogenesis and Molecular Medicine, The University of Chicago, Chicago, IL 60637, USA; E-Mail: rclark1@uchicago.edu; 3 Department of Human Genetics, The University of Chicago, Chicago, IL 60637, USA; E-Mail: rbainer@uchicago.edu

**Keywords:** prostate, metastasis, bone, chemokines

## Abstract

Bone is the most common site for metastasis in human prostate cancer patients. Skeletal metastases are a significant cause of morbidity and mortality and overall greatly affect the quality of life of prostate cancer patients. Despite advances in our understanding of the biology of primary prostate tumors, our knowledge of how and why secondary tumors derived from prostate cancer cells preferentially localize bone remains limited. The physiochemical properties of bone, and signaling molecules including specific chemokines and their receptors, are distinct in nature and function, yet play intricate and significant roles in prostate cancer bone metastasis. Examining the impact of these facets of bone metastasis *in vivo* remains a significant challenge, as animal models that mimic the natural history and malignant progression clinical prostate cancer are rare. The goals of this article are to discuss (1) characteristics of bone that most likely render it a favorable environment for prostate tumor cell growth, (2) chemokine signaling that is critical in the recruitment and migration of prostate cancer cells to the bone, and (3) current animal models utilized in studying prostate cancer bone metastasis. Further research is necessary to elucidate the mechanisms underlying the extravasation of disseminated prostate cancer cells into the bone and to provide a better understanding of the basis of cancer cell survival within the bone microenvironment. The development of animal models that recapitulate more closely the human clinical scenario of prostate cancer will greatly benefit the generation of better therapies.

## 1. Introduction

Despite significant advances in our understanding of the molecular and cellular changes involved in the initiation and progression of cancer, there has not been a substantial reduction in proportionate cancer deaths. In 2007, according to the most recent global data available, there were an estimated 12 million new cancer cases and 7.6 million deaths worldwide [1]. Ninety percent of these deaths can be attributed to metastatic disease, which is typically difficult to cure by conventional therapies (surgery, radiation and chemotherapy) [2]. These dismal statistics are largely due to the challenges posed by the complexity and heterogeneity of metastatic carcinomas, especially with regard to tumor dissemination and organ-specific colonization.

**Figure 1.**
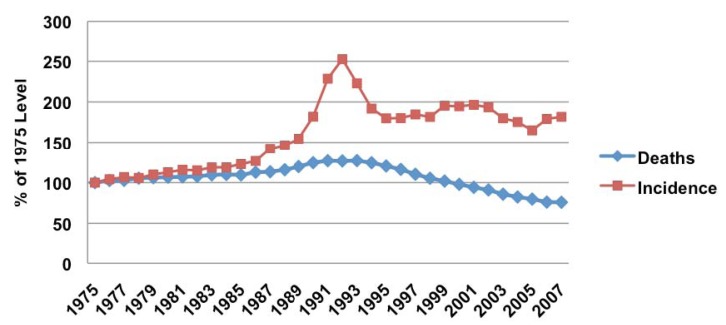
Relative changes in incidence and prostate cancer deaths in the United States from 1975 to 2007. Data from [11].

Prostate cancer is the most commonly diagnosed noncutaneous cancer, and the second most common cause of cancer-related death among men in the United States. In 2010, an estimated 217,730 men will receive a diagnosis of prostate cancer, with an estimated lifetime disease incidence of 20% [3]. Due in large part to the introduction of prostate cancer screening with prostate-specific antigen (PSA) in the mid-1980s, prostate cancer diagnoses have increased over the past four decades (Figure 1). Subsequently, a small increase in prostate cancer-specific mortality was noted secondary to increased diagnoses and attribution of deaths to the disease. Since 1993, the overall mortality rate of prostate cancer has been steadily declining, which likely reflects the combination of improved disease detection at earlier stages and advances in locoregional treatment such as surgery and radiation therapies.

Metastatic progression contributes to the majority of the morbidity and mortality associated with prostate cancer. While only 4% of men with prostate cancer have metastatic disease, the presence of metastases portends a poor prognosis with a five-year survival rate of 30% (compared with 100% for locoregional disease) [4]. In the case of disease progression, cancer cells first spread to the regional lymph nodes and then primarily to bone. The long-observed proclivity for prostate cancer cells to the bone is supported by clinical data and autopsy studies. For example, in a recent autopsy study of 1,589 patients with prostate cancer, 90% of patient with metastases had bony involvement, while only 10% of patients had only non-bony hematogenous metastases such as lung, liver, pleura and adrenal gland [5]. In addition, an inverse relationship between spine and lung metastases was noted, indicating that the two patterns of metastases may be independent.

From a clinical standpoint, the development of bone metastases is the cause of significant morbidity, including hypercalcemia, bone pain and skeletal-related events (SREs) such as pathologic fracture, spinal cord compression and palliative surgery or radiation therapy [6]. Bisphosphonate therapies have been shown to reduce SREs [7], and several bone-specific agents such as a human monoclonal antibody against the receptor activator of nuclear factor-kappa ligand (RANKL) and several endothelin-1 receptor antagonists are currently being evaluated to ameliorate osteoclast-mediated bone resorption, subsequent release of growth factors and further tumor proliferation and bone destruction [8-10].

In support of Paget's seed and soil theory, the preferential localization of prostate cancer cells to bone is not explainable by circulatory patterns or other simplified models but rather by a growth-supportive interaction between the disseminated cells and the secondary organ. Thus, we will use prostate cancer skeletal homing as our model for examining the organotropic metastatic process, considering intrinsic physical properties of bone itself, host-organ signaling characteristics, and animal models utilized to study prostate cancer bone metastasis. An improved understanding of the natural history of prostate cancer metastasis is critical for the development of targeted therapies for the treatment of this disease and for greater understanding of the metastatic cascade. To review the current knowledge about pathways of metastatic progression, this article will focus on prostate cancer given its high fidelity for a specific metastatic site: bone.

## 2. Biophysical Properties of Bone

### 2.1. The Long Bones as a Superior Metastatic Niche

In prostate cancer, disseminated tumor cells circulate through the bloodstream and ultimately localize to the long bones, where they develop into metastatic lesions. The long bones are generally defined as the set of bones that are longer than they are wide, such as the femurs and ribs. The long bones are primarily comprised of a mineralized matrix of type 1 collagen, which is organized into a dense outer layer of cortical bone that provides structural rigidity and protection to the interior trabecular bone. In contrast to cortical bone, trabecular bone is highly vascularized and contains the bone marrow, which is metabolically active and contains a variety of cell types central to hematopoiesis and the lymphatic system [12]. A number of physical features likely combine to make the long bones a comparatively permissive environment for disseminated prostate tumor cells, and contributes to the high rate of skeletal metastases in prostate cancer.

Although disseminated tumor cells are frequently present throughout the trabecular region [13], most do not go on to develop into metastases. Instead, the majority of metastatic lesions are localized to the red marrow within the metaphysis, a wide section of bone near the epiphyseal plate that is surrounded by fenestrated blood vessels known as sinusoids. The epithelia of sinusoids are permissive of lymphatic and hematopoietic cells, and provide a natural entry point for disseminated cancer cells into the trabecular bone. This entry is assisted by a number of tethering proteins that are constitutively expressed in the sinusoid epithelium, such as vascular cell adhesion molecule 1 (VCAM-1), as well as decreased rates of blood flow within the sinusoids themselves, which allow prostate cancer cells to adhere and localize to these sites [14,15]. The importance of sinusoids to metastasis is further underscored by the observation that metastatic foci in bone frequently develop at sites rich in sinusoids [16].

### 2.2. Bone Remodeling and Metastatic Growth

Bones are highly dynamic tissues that experience constant turnover through the mutual contributions of specialized bone cells known as osteoblasts and osteoclasts. Osteoblasts are derived from bone precursor cells, which differentiate from mesenchymal stem cells within the marrow and initially generate large amounts of collagen matrix proteins. Thereafter, they either undergo apoptosis and are incorporated into the existing osteoid that makes up the cortical bone, or in some cases they can be engulfed by the bone to become osteocytes. These osteocytes are thought to be able to interact with active osteoblasts in a paracrine manner to further direct bone remodeling. In contrast, osteoclasts are derived from the monocytic lineage and are primarily responsible for bone resorption. Under normal remodeling circumstances, osteoclasts dissolve osteiod, releasing growth factors trapped in the bone matrix that promote the production of new osteoblasts to further remodel the bone as needed.

Numerous growth factors, such as transforming growth factor-beta (TGF-β), and insulin-like growth factor 1 (IGF1), are released during osteoclastic bone resorption and may be co-opted to promote tumor growth, although a direct link between bone resorption and metastatic tumor growth has not yet been established. An increasing body of evidence from mouse models using breast cancer cells suggests that blocking bone resorption with bisphosphonates can reduce skeletal tumor burden by slowing or inhibiting the growth of existing lesions, and may induce apoptosis in some bone metastases [16-18]. It has been further suggested that bone resorption may suppress the ability of immune cells to target disseminated cancer cells, specifically through the release of TGF-β and consequent inhibition of T-cell and natural killer cell activity [19]. At the same time, tumor cells can secrete growth factors that directly stimulate the growth and differentiation of osteoblasts, including endothelin-1, TGF-β, insulin-like growth factors, fibroblast growth factors, and platelet-derived growth factor (PDGF) (reviewed in [20]). Finally, factors secreted by adipocytes in the bone marrow, such as tumor necrosis factor-alpha (TNF-α), interleukin-6 (IL-6) and Leptin, may indirectly contribute to metastatic growth in bone by stimulating osteoclastic resorption [12]. Taken together, the above phenomena suggest a cyclical mechanism known as the ‘vicious cycle’, wherein metastatic prostate cancer cells secrete factors to stimulate osteoclastic resorption. This then in turn releases growth factors that further support tumor proliferation and protect tumor cells from effective immune response, in parallel to direct tumor-mediated osteoblastic proliferation.

### 2.3. Osteomimicry

It has been proposed that in order for disseminated cancer cells to fully exploit the bone metastatic niche and successfully proliferate into metastatic lesions, those cells must acquire a “bone-like phenotype” that effectively promotes localization and proliferation [21]. This has been supported by the observation that prostate cancer cells in culture may display properties associated with bone tissue, and that sublines of prostate cancer cells cultivated *in vivo* can acquire characteristic gene expression signatures that correlate with both osteoclastic development and metastatic phenotype [21]. In some cases cell lines have been shown to mimic osteoblastic phenotypes by overexpressing bone matrix proteins that are normally exclusive to bone, such as osteonectin and osteopontin, or by secreting factors common to osteoblasts such as beta-2 microglobulin (β2M) and receptor activator of NF-κB ligand (RANKL) [22-25]. In other cases, prostate cancer cells can overexpress genes responsible for osteoclast differentiation and osteoblast mineralization, such as parathyroid hormone-related protein (PTHrP) and inhibitor of DNA binding-1 (Id-1) [26]. The osteomimetic phenotype among some prostate cancer cell lines is so pronounced that they can stimulate the production of mineralized bone in cultured osteoblast cells *in vitro* [21,25].

## 3. Chemokines and Their Receptors

**Figure 2.**
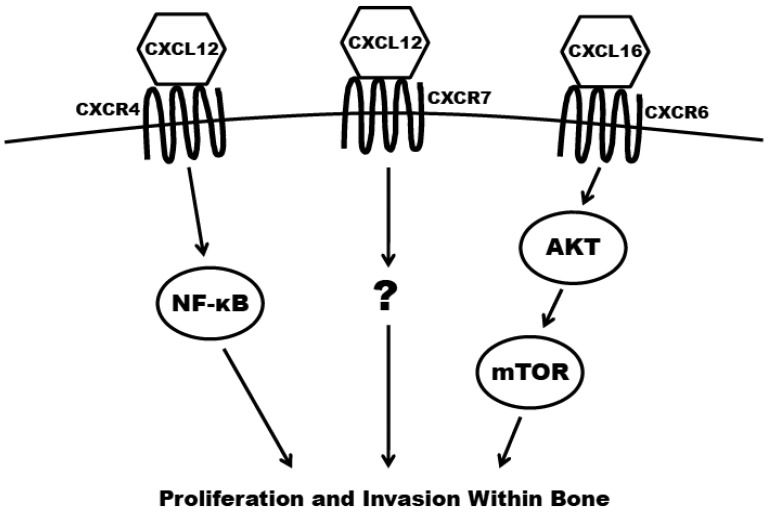
Cytokine signaling through their cognate receptors results in promotion of prostate cancer metastasis formation in the bone (Prepared by author Robert J. Clark).

The concept of chemotaxis, the directed migration of a cell toward the source of a secreted protein signal, has been most classically studied in the context of leukocyte trafficking to the site of infection. Chemokines are a class of chemotaxic signals that are considered pro-inflammatory, meaning they recruit immune cells to sites of injury or infection and promote angiogenesis and cellular proliferation at those sites. Chemokine binding to their corresponding seven transmembrane-domain G-protein-coupled receptors causes activation of signal transduction networks leading to chemotaxis. Chemokines and their receptors are classified and named based on the position of the first N-terminal cysteines (C, CC, CXC, CX_3_C) (reviewed in [27]). The receptors have been implicated in the migration of other cell types, including breast [28], lung [29] and prostate cancers to secondary sites in the bone. In the case of prostate cancer dissemination, or homing, to the bone, CXCR4 (CXC receptor 4), CXCR7 and CXCR6 are believed to have the greatest impact (Figure 2) and are discussed in the remainder of this section.

### 3.1. CXCR4

CXCR4 is most widely studied for its role in both pre-pro B-cell survival [30] and as an essential cofactor in T cell infection by human immunodeficiency virus [31]. However, it has also been shown to play a key role in tumorigenesis and metastasis of prostate and other cancers. The ligand for CXCR4 is CXCL12 (also known as stromal derived factor 1; SDF1) and is highly expressed at sites of prostate cancer metastasis including lymph nodes, bone, lungs and liver. CXCR4 is expressed in primary prostate tumors and prostate metastases at a higher level than in normal prostate tissue [32,33]. It is also present in high levels on the surface of commonly utilized prostate cancer cell lines, including PC3, LNCaP and DU145 [32,34]. The expression of CXCR4 has been shown to be positively regulated by androgen receptor (AR) signaling, the critical pathway in the survival and proliferation of prostate cells. AR activation induces the transcription of Krueppel-like factor 5 (KLF5), another transcription factor that in turn promotes the expression of CXCR4 [35]. CXCR4 has also been shown to play an important role in prostate cancer cell adhesion. Treatment of prostate cancer cells with CXCL12 increase their adhesion to a bone-marrow derived endothelial cell monolayer in culture [36]. Kukreja *et al.* demonstrated that the CXCR4/CXCL12 mediated adhesion occurred at least partially through the NF-κB pathway [37]. In addition, activation of CXCR4 by CXCL12 also causes prostate cancer cells to upregulate the expression of alpha(v)beta(3) integrins, surface receptors that mediate cell-cell and cell-extracellular matrix interactions. This integrin upregulation leads to increased *in vitro* adhesion and invasiveness of prostate cancer cells [38,39]. Furthermore, the knockdown of CXCR4 leads to a decrease in angiogenesis, lymphangiogenesis and vascular endothelial growth factor (VEGF) expression, and an increase in apoptosis in xenograft models [40]. Xing *et al.* showed that knockdown also significantly decreased overall bone metastasis *in vivo* [41].

### 3.2. CXCR7

CXCR7 (also known as receptor dog cDNA 1; RDC1) is a more recently discovered chemokine receptor that also preferentially binds CXCL12 [42]. It is still unclear if CXCR7 is expressed at all on any class of leukocyte in adult mammals [43,44]. Accordingly, some debate still exists as to whether or not CXCR7 primarily acts as a decoy (non-signaling) receptor [45,46]. There is evidence, however, that CXCL12 mediated signaling can promote a metastatic phenotype. CXCR7 expression is higher in malignant cell lines *versus* non transformed counterparts [47]. Also, the receptor is more highly expressed in prostate metastases (especially those to bone) compared to primary tumors seen in clinical specimens [48]. Overexpression of CXCR7 in PC3 and LNCaP cells results in increased proliferation, adhesion and invasion *in vitro*. Furthermore, CXCR7 overexpression increases the production of IL-8 and VEGF, two factors known to be involved in the formation of bone metastases [48]. Finally, overexpression of CXCR7 in rhabdomyosarcoma cell lines significantly increased their metastasis to the bone in xenograft models [49].

### 3.3. CXCR6

CXCR6 (previously termed “Bonzo”) is expressed on polarized subsets of T cells and is at least partially responsible for their homing to sites of inflammation [50]. The primary ligand for CXCR6 is CXCL16, a molecule that can be found both membrane-bound and in a soluble form. CXCL16 is predominantly expressed by circulating leukocytes [51] but is also found at high levels in the bone marrow [52,53]. CXCR6 is highly expressed in prostate cancer cell lines [53]. In patient tissue samples of prostate cancer, the expression of the receptor increases proportionately to the Gleason score. *In vitro*, the overexpression of CXCR6 leads to a significant increase in the migration and invasion of LNCaP, PC3 and DU145 cells using transwell assays [54,55]. Finally, Wang and colleagues demonstrated that the activation of CXCR6 by CXCL16 led to an increase in signaling of the Akt/mammalian target of rapamycin (mTOR) pathway. Furthermore, treatment with rapamycin, a specific inhibitor of mTOR, significantly inhibited proliferation and invasion of CXCL16 treated prostate cancer cells [55].

### 3.4. Summary

Taken together, these data present strong evidence of chemokines and their receptors playing a critical role in the homing of prostate cancer to bone. Interestingly, each of the discussed pairs can be found in a variety of tissues and therefore does not exclude other organs as potential sites of prostate cancer metastasis. We speculate that their combined activity, along with known molecules expressed in prostate cancer cells that promote cancer-bone interactions such as matrix metalloproteinases (MMPs) (reviewed in [56]), RANKL [57], and PHTrP [58], allow for a clearer picture of the signaling events that promote such organotropism. These findings beg additional functional evidence for the importance of chemokine/chemokine receptor signaling in prostate cancer bone metastasis is needed. Most of the *in vitro* work is done with cell lines that do not express AR or PSA while more clinically relevant lines are readily available. More importantly, few of these studies have been translated in *in vivo* models. Further experiments directly assessing metastasis utilizing appropriate animal models are necessary to show the functionality of chemotaxis and other key events in the progression of prostate cancer metastasis to the bone.

## 4. Animal Models of Prostate Cancer: Metastasis to Bone

To fully understand the mechanisms by which prostate cancer cells metastasize, models that closely resemble the pathology and sequential order of human prostate metastatic disease are necessary. While there are several good animal models of primary prostate cancers that mimic the pathology of human disease, at present there is no model that sufficiently recapitulates human prostate cancer as it metastasizes mostly to the bone. There are several prostate cancer cell lines that form primary tumors, but will not metastasize to bone following orthotopic transplantation into the prostate. Some widely used models of metastatic prostate cancer produce metastases that primarily localize to the lymph nodes or lung and only produce sporadic bone metastases. Moreover, injection of human prostate cells into immunocompromised animals has the inherent limitation of downplaying potential interactions between prostate cancer cells and immune cells. This interaction might be important for site-specific lodging of prostate cancer cells in bone, and should be considered in these studies. An overview of selected *in vivo* models is described below and in Table 1.

### 4.1. Spontaneous and Experimental Metastasis Models

*In vivo* approaches to studying metastasis can be generally grouped into spontaneous or experimental models. In the spontaneous model, tumor cells are injected at a primary site (e.g., orthotopic site or possibly subcutaneous in the flank) and after primary tumors form, cells follow the entire metastatic cascade from invading through the basement membrane, surviving in the circulation, extravasating, and developing as metastatic foci at a secondary site. In an experimental metastasis model, cancer cells are injected into the bloodstream, either by intracardiac or tail vein injection, bypassing earlier steps in the cascade. To specifically examine colonization of the bone microenvironment, prostate cancer cells can be injected directly into the bone. Although this does not test metastasis *per se*, it can be useful to further our understanding of cancer cell-bone microenvironment interactions. Unfortunately, the majority of prostate cancer xenograft models do not form bone metastases efficiently.

Several studies have been performed utilizing human prostate cancer cells transplanted into immunocompromised mice or rats, although the majority of these cells will not inherently metastasize to bone. Thalmann *et al.* developed sublines of C4-2 (androgen-insensitive LNCaP derivative) cells that are derived from bone metastases in nude mice following injection of C4-2 cells. In a spontaneous metastasis assay, all four derived sublines secreted PSA and metastasized to the bone following subcutaneous inoculation. Interestingly, these sublines formed tumors faster than the parental C4-2 cells. Moreover, when injected subcutaneously, a large fraction of these sublines led to paraplegia of the mouse (as early as five weeks) more rapidly than injection of control C4-2 cells [59]. Similar results were obtained in an experimental metastasis model utilizing CWR22 prostate cancer cells. CWR22 cells closely resemble early-stage prostate cancer cells in that they secrete PSA, are relatively slow-growing, and have both androgen-dependent and androgen-independent stages. Andresen *et al.* demonstrated the formation of osteosclerotic bone lesions three to four weeks following intra-tibial injection of CWR22 cells into Sprague Dawley immunodeficient rats. These lesions appeared fully mineralized and osteoblastic in nature, similar to human disease [60].

### 4.2. R3327 Dunning Model

Prostate cancer and bone metastasis has been extensively studied using rats as a model system, primarily because the size of the bone is greater than that of a mouse, allowing for easier handling, processing and analyses of the bone. In the early 1960s, a spontaneous prostate tumor was observed in a 22 month-old Copenhagen male rat. Grafts were taken from this tumor (termed “R3327”) and were subsequently serially transplanted subcutaneously into intact or castrated rats. This gave rise to several biochemically distinct sublines, including the androgen-insensitive and aggressive Mat-Ly-Lu cell line [61,62]. These cells form osteoblastic lesions in Copenhagen rats upon intra-tibial and intracardiac injection or using tail vein injection with simultaneous clamping of the lower caval vein. Mat-Ly-Lu cells are highly aggressive and lead to increased morbidity and mortality within one month of injection (for a comprehensive review on R3327 cell lines refer to [63]).

### 4.3. Ras Signaling

More recently, cell lines modified by overexpression or knockdown of molecules potentially involved in prostate cancer bone metastasis have been created. These cells have subsequently been utilized *in vivo* to determine the impact of specific signaling pathways on bone metastasis. For example, overexpression of RalGEF, a downstream effector of Ras signaling, is sufficient to enable DU145 cells to form bone metastases following intracardiac injection. Conversely, inhibiting RalGEF signaling through shRNA in PC3 cells led to inhibition of growth of bone metastases (as determined by a relative decrease in bioluminescence signal) in the otherwise metastatic PC3 cell line when injected into the left ventricle [64].

### 4.4. Transgenic Mice

Unfortunately, while there are several transgenic animal models of prostate cancer that closely resemble the pathology of human disease, it is rare that the primary tumors in these contexts metastasize to the bone. For example, a mouse model containing a hemizygous deletion of both *NKX3.1* and *PTEN* develops high-grade PIN lesions by six months of age and invasive adenocarcinoma after 12 months of age. While 25% of these mice between aged 12–15 months had lymph node metastases, none had visible metastases to any other organ, including bone [65]. Similarly, mice containing a conditional deletion of the tumor suppressor proteins p53 and Rb in the prostate epithelium also develop invasive adenocarcinomas with overt liver and lung metastases apparent by 200 days. Lymph node metastases were also observed, although no mice had apparent bone metastasis. Interestingly, osteopontin, which is frequently overexpressed and associated with human prostate cancer bone metastases, was found to be upregulated in p53/Rb conditional knockout mice [66,67]. One of the most aggressive transgenic mouse models of prostate cancer, the TRAMP mouse model, develops prostate cancer at a relatively young age. In these mice, the SV40 early genes expression is driven specifically in the prostate epithelium, and induces the development of invasive prostatic adenocarcinoma by age 18 weeks. By 28 weeks of age, all mice had lung or lymph node metastasis. While it is possible for TRAMP mice to develop bone metastases, it is rare [68,69]. The low frequency of bone metastasis observed across diverse mouse models of prostate cancer such as these further underscores the importance of developing an *in vivo* system that can be readily utilized to elucidate the mechanisms involved in human prostate cancer metastasis to the bone.

### 4.5. Summary

To fully understand the mechanisms by which clinical prostate cancer preferentially form skeletal metastases, we need to develop models that more closely resemble the pathology and sequential order of clinical disease. At present, there is no animal model of prostate cancer that sufficiently recapitulates human prostate cancer as it metastasizes mostly to the bone. There are several prostate cancer cell lines that form primary tumors, but will not metastasize to bone. Other widely used models of metastatic prostate cancer produce metastases that primarily localize to the lymph nodes or lung and only produce sporadic bone metastases. Moreover, the injection of human prostate cells into immunocompromised animals inherently overlooks the importance of the immune compartment in bone metastasis formation. This interaction might be important for site-specific lodging of prostate cancer cells in bone, and should be considered in these studies.

The lack of animal models closely resembling that of human prostate disease is a major gap in the field and limits the extent to which prostate cancer metastasis can be studied. In particular, the field would benefit from a spontaneous or transgenic model that can be used to study the entire metastatic cascade beginning with a primary prostate tumor and ending with colonization in the bone. Moreover, an animal model that recapitulates the osteomimetic nature of certain prostate cancer cells and/or transgenic models of prostate cancer that manipulate the key cytokine signaling pathways could potentially more closely mimic human disease.

## 5. Concluding Remarks

As clinical detection and management of locoregional prostate cancer has become more sophisticated, research attention has shifted to its metastasis. Considering that the overwhelming majority of advanced prostate cancers will colonize the bone, there is specific interest in understanding the nuances of that organotropic spread. Thus far, chemokine/chemokine receptor interactions that direct prostate cancer homing as well as characteristics of bone that make it ideally suited to host disseminated cells have been shown to be critical to this process. However, animal models that can recapitulate the natural progression from primary tumor to skeletal metastases simply do not exist. Therefore, new animal models that more closely mimic the evolution of prostate cancer in human patients would be critical to further our understanding of how and why disseminated prostate cancer cells preferentially colonize the bone. Furthermore, such advances would provide much needed insight toward the development of potential therapeutic options for this highly prevalent disease.

## Figures and Tables

**Table 1.** Overview of Selected Animal Models of Prostate Cancer.

**Model**	**Method**	**Metastasize to Bone?**
NKX3.1+/-, PTEN+/-, p53/Rb conditional knockout, TRAMP	Transgenic mice	Rare
Mat-Ly-Lu	Injected intracardiac, intratibial, or tail vein	Osteoblastic
C4-2 androgen-insensitive sublines	Injected subcutaneously; Athymic nude mice	Osteoblastic; paraplegia
CWR22	Injected intratibial; Immunodeficient rats	Osteoblastic
RalGEF overexpression (Ras pathway activation)	Injected intracardiac; Athymic nude mice	Osteolytic; osteoblasts and osteoclasts

## References

[1] Garcia M., Jemal A., Ward E.M., Center M.M., Hao Y., Siegel R.L., Thun M.J. (2007). Global Cancer Facts & Figures 2007.

[2] Hanahan D., Weinberg R.A. (2000). The hallmarks of cancer. Cell.

[3] Jemal A., Siegel R., Xu J., Ward E. (2010). Cancer statistics, 2010. CA Cancer J. Clin..

[4] National Cancer Institute Surveillance Research Program Cancer Statistics Branch (2010). Surveillance, Epidemiology, and End Results (SEER) Program Populations (1969-2007).

[5] Bubendorf L., Schopfer A., Wagner U., Sauter G., Moch H., Willi N., Gasser T.C., Mihatsch M.J. (2000). Metastatic patterns of prostate cancer: An autopsy study of 1,589 patients. Hum. Pathol..

[6] Coleman R.E. (2001). Metastatic bone disease: Clinical features, pathophysiology and treatment strategies. Cancer Treat. Rev..

[7] Saad F., Gleason D.M., Murray R., Tchekmedyian S., Venner P., Lacombe L., Chin J.L., Vinholes J.J., Goas J.A., Zheng M. (2004). Long-term efficacy of zoledronic acid for the prevention of skeletal complications in patients with metastatic hormone-refractory prostate cancer. J. Natl. Cancer Inst..

[8] Fizazi K., Bosserman L., Gao G., Skacel T., Markus R. (2009). Denosumab treatment of prostate cancer with bone metastases and increased urine n-telopeptide levels after therapy with intravenous bisphosphonates: Results of a randomized phase ii trial. J. Urol..

[9] James N.D., Caty A., Payne H., Borre M., Zonnenberg B.A., Beuzeboc P., McIntosh S., Morris T., Phung D., Dawson N.A. (2010). Final safety and efficacy analysis of the specific endothelin a receptor antagonist zibotentan (zd4054) in patients with metastatic castration-resistant prostate cancer and bone metastases who were pain-free or mildly symptomatic for pain: A double-blind, placebo-controlled, randomized phase ii trial. BJU Int..

[10] Nelson J.B., Love W., Chin J.L., Saad F., Schulman C.C., Sleep D.J., Qian J., Steinberg J., Carducci M. (2008). Phase 3, randomized, controlled trial of atrasentan in patients with nonmetastatic, hormone-refractory prostate cancer. Cancer.

[b11-cancers-03-00478] 11.Surveillance, epidemiology, and end results (seer) program (http://www.Seer.Cancer.Gov). Seer*stat database: Mortality - all cod, aggregated with state, total U.S. (1969-2007). In National Cancer Institute, DCCPS, Surveillance Research Program, Cancer Statistics Branch, released June 2010, based on the November 2009 submission. Underlying mortality data provided by NCHS (http://www.cdc.gov/nchs).

[12] Bussard K.M., Gay C.V., Mastro A.M. (2008). The bone microenvironment in metastasis; what is special about bone?. Cancer Metastasis Rev..

[13] Klein C.A. (2003). The systemic progression of human cancer: A focus on the individual disseminated cancer cell--the unit of selection. Adv. Cancer Res..

[14] Scott L.J., Clarke N.W., George N.J., Shanks J.H., Testa N.G., Lang S.H. (2001). Interactions of human prostatic epithelial cells with bone marrow endothelium: Binding and invasion. Br. J. Cancer.

[15] Jacobsen K., Kravitz J., Kincade P.W., Osmond D.G. (1996). Adhesion receptors on bone marrow stromal cells: *In vivo* expression of vascular cell adhesion molecule-1 by reticular cells and sinusoidal endothelium in normal and gamma-irradiated mice. Blood.

[16] Sasaki A., Boyce B.F., Story B., Wright K.R., Chapman M., Boyce R., Mundy G.R., Yoneda T. (1995). Bisphosphonate risedronate reduces metastatic human breast cancer burden in bone in nude mice. Cancer Res..

[17] Fournier P.G., Stresing V., Ebetino F.H., Clezardin P. (2010). How do bisphosphonates inhibit bone metastasis *in vivo*?. Neoplasia.

[18] Sun M., Iqbal J., Singh S., Sun L., Zaidi M. (2010). The crossover of bisphosphonates to cancer therapy. Ann. N. Y. Acad. Sci..

[19] Fournier P.G., Chirgwin J.M., Guise T.A. (2006). New insights into the role of t cells in the vicious cycle of bone metastases. Curr. Opin. Rheumatol..

[20] Mundy G.R. (2002). Metastasis to bone: Causes, consequences and therapeutic opportunities. Nat. Rev. Cancer..

[21] Koeneman K.S., Yeung F., Chung L.W. (1999). Osteomimetic properties of prostate cancer cells: A hypothesis supporting the predilection of prostate cancer metastasis and growth in the bone environment. Prostate.

[22] Huang W.C., Wu D., Xie Z., Zhau H.E., Nomura T., Zayzafoon M., Pohl J., Hsieh C.L., Weitzmann M.N., Farach-Carson M.C., Chung L.W. (2006). Beta2-microglobulin is a signaling and growth-promoting factor for human prostate cancer bone metastasis. Cancer Res..

[23] Josson S., Matsuoka Y., Chung L.W., Zhau H.E., Wang R. (2010). Tumor-stroma co-evolution in prostate cancer progression and metastasis. Semin. Cell Dev. Biol..

[24] Graham T.R., Agrawal K.C., Abdel-Mageed A.B. (2010). Independent and cooperative roles of tumor necrosis factor-alpha, nuclear factor-kappab, and bone morphogenetic protein-2 in regulation of metastasis and osteomimicry of prostate cancer cells and differentiation and mineralization of mc3t3-e1 osteoblast-like cells. Cancer Sci..

[25] Lecrone V., Li W., Devoll R.E., Logothetis C., Farach-Carson M.C. (2000). Calcium signals in prostate cancer cells: Specific activation by bone-matrix proteins. Cell Calcium.

[26] Yuen H.F., Chiu Y.T., Chan K.K., Chan Y.P., Chua C.W., McCrudden C.M., Tang K.H., El-Tanani M., Wong Y.C., Wang X., Chan K.W. (2010). Prostate cancer cells modulate osteoblast mineralisation and osteoclast differentiation through id-1. Br. J. Cancer.

[27] Vandercappellen J., Van Damme J., Struyf S. (2008). The role of cxc chemokines and their receptors in cancer. Cancer Lett..

[28] Muller A., Homey B., Soto H., Ge N., Catron D., Buchanan M.E., McClanahan T., Murphy E., Yuan W., Wagner S.N., Barrera J.L., Mohar A., Verastegui E., Zlotnik A. (2001). Involvement of chemokine receptors in breast cancer metastasis. Nature.

[29] Nakamura E.S., Koizumi K., Kobayashi M., Saitoh Y., Arita Y., Nakayama T., Sakurai H., Yoshie O., Saiki I. (2006). Rankl-induced ccl22/macrophage-derived chemokine produced from osteoclasts potentially promotes the bone metastasis of lung cancer expressing its receptor ccr4. Clin. Exp. Metastasis.

[30] Tokoyoda K., Egawa T., Sugiyama T., Choi B.I., Nagasawa T. (2004). Cellular niches controlling b lymphocyte behavior within bone marrow during development. Immunity.

[31] Feng Y., Broder C.C., Kennedy P.E., Berger E.A. (1996). Hiv-1 entry cofactor: Functional cdna cloning of a seven-transmembrane, g protein-coupled receptor. Science.

[32] Sun Y.X., Wang J., Shelburne C.E., Lopatin D.E., Chinnaiyan A.M., Rubin M.A., Pienta K.J., Taichman R.S. (2003). Expression of cxcr4 and cxcl12 (sdf-1) in human prostate cancers (pca) in vivo. J. Cell Biochem..

[33] Akashi T., Koizumi K., Tsuneyama K., Saiki I., Takano Y., Fuse H. (2008). Chemokine receptor cxcr4 expression and prognosis in patients with metastatic prostate cancer. Cancer Sci..

[34] Chetram M.A., Odero-Marah V., Hinton C.V. (2010). Loss of pten permits cxcr4-mediated tumorigenesis through erk1/2 in prostate cancer cells. Mol. Cancer Res..

[35] Frigo D.E., Sherk A.B., Wittmann B.M., Norris J.D., Wang Q., Joseph J.D., Toner A.P., Brown M., McDonnell D.P. (2009). Induction of kruppel-like factor 5 expression by androgens results in increased cxcr4-dependent migration of prostate cancer cells *in vitro*. Mol. Endocrinol..

[36] Taichman R.S., Cooper C., Keller E.T., Pienta K.J., Taichman N.S., McCauley L.K. (2002). Use of the stromal cell-derived factor-1/cxcr4 pathway in prostate cancer metastasis to bone. Cancer Res..

[37] Kukreja P., Abdel-Mageed A.B., Mondal D., Liu K., Agrawal K.C. (2005). Up-regulation of cxcr4 expression in pc-3 cells by stromal-derived factor-1alpha (cxcl12) increases endothelial adhesion and transendothelial migration: Role of mek/erk signaling pathway-dependent nf-kappab activation. Cancer Res..

[38] Engl T., Relja B., Marian D., Blumenberg C., Muller I., Beecken W.D., Jones J., Ringel E.M., Bereiter-Hahn J., Jonas D., Blaheta R.A. (2006). Cxcr4 chemokine receptor mediates prostate tumor cell adhesion through alpha5 and beta3 integrins. Neoplasia.

[39] Sun Y.X., Fang M., Wang J., Cooper C.R., Pienta K.J., Taichman R.S. (2007). Expression and activation of alpha v beta 3 integrins by sdf-1/cxc12 increases the aggressiveness of prostate cancer cells. Prostate.

[40] Porvasnik S., Sakamoto N., Kusmartsev S., Eruslanov E., Kim W.J., Cao W., Urbanek C., Wong D., Goodison S., Rosser C.J. (2009). Effects of cxcr4 antagonist ctce-9908 on prostate tumor growth. Prostate.

[41] Xing Y., Liu M., Du Y., Qu F., Li Y., Zhang Q., Xiao Y., Zhao J., Zeng F., Xiao C. (2008). Tumor cell-specific blockade of cxcr4/sdf-1 interactions in prostate cancer cells by htert promoter induced cxcr4 knockdown: A possible metastasis preventing and minimizing approach. Cancer Biol. Ther..

[42] Balabanian K., Lagane B., Infantino S., Chow K.Y., Harriague J., Moepps B., Arenzana-Seisdedos F., Thelen M., Bachelerie F. (2005). The chemokine sdf-1/cxcl12 binds to and signals through the orphan receptor rdc1 in t lymphocytes. J. Biol. Chem..

[43] Berahovich R.D., Zabel B.A., Penfold M.E., Lewen S., Wang Y., Miao Z., Gan L., Pereda J., Dias J., Slukvin II, McGrath K.E., Jaen J.C., Schall T.J. (2010). Cxcr7 protein is not expressed on human or mouse leukocytes. J. Immunol..

[44] Borge M., Nannini P.R., Galletti J.G., Morande P.E., Avalos J.S., Bezares R.F., Giordano M., Gamberale R. (2010). Cxcl12-induced chemotaxis is impaired in t cells from patients with zap-70-negative chronic lymphocytic leukemia. Haematologica.

[45] Naumann U., Cameroni E., Pruenster M., Mahabaleshwar H., Raz E., Zerwes H.G., Rot A., Thelen M. (2010). Cxcr7 functions as a scavenger for cxcl12 and cxcl11. PLoS One.

[46] Rajagopal S., Kim J., Ahn S., Craig S., Lam C.M., Gerard N.P., Gerard C., Lefkowitz R.J. (2010). Beta-arrestin- but not g protein-mediated signaling by the “Decoy” Receptor cxcr7. Proc. Natl. Acad. Sci. USA.

[47] Burns J.M., Summers B.C., Wang Y., Melikian A., Berahovich R., Miao Z., Penfold M.E., Sunshine M.J., Littman D.R., Kuo C.J., Wei K., McMaster B.E., Wright K., Howard M.C., Schall T.J. (2006). A novel chemokine receptor for sdf-1 and i-tac involved in cell survival, cell adhesion, and tumor development. J. Exp. Med..

[48] Wang J., Shiozawa Y., Wang Y., Jung Y., Pienta K.J., Mehra R., Loberg R., Taichman R.S. (2008). The role of cxcr7/rdc1 as a chemokine receptor for cxcl12/sdf-1 in prostate cancer. J. Biol. Chem..

[49] Grymula K., Tarnowski M., Wysoczynski M., Drukala J., Barr F.G., Ratajczak J., Kucia M., Ratajczak M.Z. (2010). Overlapping and distinct role of cxcr7-sdf-1/itac and cxcr4-sdf-1 axes in regulating metastatic behavior of human rhabdomyosarcomas. Int. J. Cancer.

[50] Kim C.H., Kunkel E.J., Boisvert J., Johnston B., Campbell J.J., Genovese M.C., Greenberg H.B., Butcher E.C. (2001). Bonzo/cxcr6 expression defines type 1-polarized t-cell subsets with extralymphoid tissue homing potential. J. Clin. Invest..

[51] Tabata S., Kadowaki N., Kitawaki T., Shimaoka T., Yonehara S., Yoshie O., Uchiyama T. (2005). Distribution and kinetics of sr-psox/cxcl16 and cxcr6 expression on human dendritic cell subsets and cd4+ t cells. J. Leukoc. Biol..

[52] Nakayama T., Hieshima K., Izawa D., Tatsumi Y., Kanamaru A., Yoshie O. (2003). Cutting edge: Profile of chemokine receptor expression on human plasma cells accounts for their efficient recruitment to target tissues. J. Immunol..

[53] Hu W., Zhen X., Xiong B., Wang B., Zhang W., Zhou W. (2008). Cxcr6 is expressed in human prostate cancer in vivo and is involved in the in vitro invasion of pc3 and lncap cells. Cancer Sci..

[54] Lu Y., Wang J., Xu Y., Koch A.E., Cai Z., Chen X., Galson D.L., Taichman R.S., Zhang J. (2008). Cxcl16 functions as a novel chemotactic factor for prostate cancer cells in vitro. Mol. Cancer Res..

[55] Wang J., Lu Y., Koch A.E., Zhang J., Taichman R.S. (2008). Cxcr6 induces prostate cancer progression by the akt/mammalian target of rapamycin signaling pathway. Cancer Res..

[56] Lokeshwar B.L. (1999). Mmp inhibition in prostate cancer. Ann. N. Y. Acad. Sci..

[57] Zhang J., Dai J., Qi Y., Lin D.L., Smith P., Strayhorn C., Mizokami A., Fu Z., Westman J., Keller E.T. (2001). Osteoprotegerin inhibits prostate cancer-induced osteoclastogenesis and prevents prostate tumor growth in the bone. J. Clin. Invest..

[58] Asadi F., Farraj M., Sharifi R., Malakouti S., Antar S., Kukreja S. (1996). Enhanced expression of parathyroid hormone-related protein in prostate cancer as compared with benign prostatic hyperplasia. Hum. Pathol..

[59] Thalmann G.N., Sikes R.A., Wu T.T., Degeorges A., Chang S.M., Ozen M., Pathak S., Chung L.W. (2000). Lncap progression model of human prostate cancer: Androgen-independence and osseous metastasis. Prostate.

[60] Andersen C., Bagi C.M., Adams S.W. (2003). Intra-tibial injection of human prostate cancer cell line cwr22 elicits osteoblastic response in immunodeficient rats. J. Musculoskelet Neuronal. Interact..

[61] Quarmby V.E., Beckman W.C., Cooke D.B., Lubahn D.B., Joseph D.R., Wilson E.M., French F.S. (1990). Expression and localization of androgen receptor in the r-3327 dunning rat prostatic adenocarcinoma. Cancer Res..

[62] Isaacs J.T., Isaacs W.B., Feitz W.F., Scheres J. (1986). Establishment and characterization of seven dunning rat prostatic cancer cell lines and their use in developing methods for predicting metastatic abilities of prostatic cancers. Prostate.

[63] Blouin S., Basle M.F., Chappard D. (2005). Rat models of bone metastases. Clin. Exp. Metastasis.

[64] Yin J., Pollock C., Tracy K., Chock M., Martin P., Oberst M., Kelly K. (2007). Activation of the ralgef/ral pathway promotes prostate cancer metastasis to bone. Mol. Cell Biol..

[65] Abate-Shen C., Banach-Petrosky W.A., Sun X., Economides K.D., Desai N., Gregg J.P., Borowsky A.D., Cardiff R.D., Shen M.M. (2003). Nkx3.1; pten mutant mice develop invasive prostate adenocarcinoma and lymph node metastases. Cancer Res..

[66] Zhou Z., Flesken-Nikitin A., Corney D.C., Wang W., Goodrich D.W., Roy-Burman P., Nikitin A.Y. (2006). Synergy of p53 and rb deficiency in a conditional mouse model for metastatic prostate cancer. Cancer Res..

[67] Nemoto H., Rittling S.R., Yoshitake H., Furuya K., Amagasa T., Tsuji K., Nifuji A., Denhardt D.T., Noda M. (2001). Osteopontin deficiency reduces experimental tumor cell metastasis to bone and soft tissues. J. Bone Miner Res..

[68] Gingrich J.R., Barrios R.J., Morton R.A., Boyce B.F., DeMayo F.J., Finegold M.J., Angelopoulou R., Rosen J.M., Greenberg N.M. (1996). Metastatic prostate cancer in a transgenic mouse. Cancer Res..

[69] Hsieh C.L., Xie Z., Yu J., Martin W.D., Datta M.W., Wu G.J., Chung L.W. (2007). Non-invasive bioluminescent detection of prostate cancer growth and metastasis in a bigenic transgenic mouse model. Prostate.

